# Spatio-temporal variability of jet streams over North America and North Pacific Ocean

**DOI:** 10.1038/s41598-025-21354-2

**Published:** 2025-10-24

**Authors:** Saadoun Salimi, Taha B. M. J. Ouarda

**Affiliations:** Canada Research Chair in Statistical Hydro-Climatology, Institut National de la Recherche Scientifique, Centre Eau Terre Environnement, INRS-ETE, 490 de la Couronne, Québec, QC G1K 9A9 Canada

**Keywords:** Jet stream, North America, Wavelet analysis, Seasonal variability, North Pacific Ocean, Climate sciences, Atmospheric science

## Abstract

This study examines the impact of climate change on jet stream features and their seasonal fluctuations across North America (NA) from 1984 to 2023. Maps and analyses were produced utilizing ERA5, ERA-Interim, and NCEP/NNCAR data for Temperature (T), Zonal wind (Uwnd), and Meridional wind (Vwnd). Results indicate two important places where jet streams are significantly affected by climate change: the North Pacific Ocean (NPO) and the eastern portion of North America (EPNA). The most varied jet stream trajectories in the NPO manifest during summer, but in EPNA, they peak in autumn. Jet streams are positioned lower and exhibit more velocity in winter, whereas they are situated higher and demonstrate less velocity in summer. In the last 40 years, jet streams have demonstrated cyclical patterns of 5, 7, and 10 years, exhibiting no altitude variations, while in other instances, they have shown fluctuations between 100 and 300 hectopascals in altitude. The winter and spring jet streams over the NPO ascended to elevated altitudes, diminishing variations, whereas the winter and autumn jet streams over EPNA descended, amplifying volatility. Seasonal analysis of temperature and zonal wind patterns revealed that rising temperatures were associated with increased zonal wind speeds across nearly all seasons. Concurrently, the jet stream cores exhibited a consistent upward and poleward shift toward higher latitudes. These currents convey moisture, influencing regional climatic patterns and resulting in occurrences such as atmospheric rivers. This study highlights the variable characteristics of jet streams and their essential function in regional climate.

## Introduction

A jet stream is a narrow, concentrated wind flowing horizontally in the upper troposphere or stratosphere. Wasaburo Oishi first observed jet stream in the 1920s. Rosebay discovered that in the upper troposphere during WWII^1^, and Heinrich Seilkopf later introduced the word ‘jet stream’^2^. During WWII, aviators noticed strong wind velocity fluctuations, which contributed to the broader understanding of these atmospheric currents^3^. The term’ jet stream’ is commonly used to refer to the polar jet streams in the Northern Hemisphere, mainly found in North America, Europe, and Asia during winter and they become stronger and cover larger areas during this season. They appear as elongated, high-velocity belts on meteorological maps, with speeds ranging from 25 to 30 m/s^4^, spanning 161 to 644 km in width and 1.6 to 4.8 km in thickness^5^. They peak at altitudes of 9000 m, with velocities of 241 to 482 km/h^6,7^ Rossby waves and baroclinic instability influence how jet streams meander, moving eastward in a wave pattern^8^.

Jet streams can be classified into several distinct types based on their geographic origins. The African Easterly Jet (AEJ) is a summer jet stream covering about 10,000 km^9–11^. They have distinct velocity cores that vary in position, forming the Polar Front Jet Stream (PFJET) and the Subtropical Jet Stream (STJ)^12,13^. The jet stream forms from the Earth’s angular momentum, while the PFJET derives kinetic energy from thermal contrasts^14,15^, and influences Earth’s climate by guiding extratropical cyclones and inducing atmospheric instability, leading to rainfall^16–18^.

Past studies have investigated different aspects of jet stream variability. Studies by Strong and Davis and Archer and Caldeira showed varying tendencies in jet stream velocities and positions^3,19^. Manney and Hegglin found polar movement in the Southern Hemisphere Jet Streams (SHJET) and equatorial movement in the Northern Hemisphere, with rising trends in jet stream elevations^20^. Other research found that Climate change significantly impacts the North Atlantic jet stream^21,22^ and the North Pacific jet’s strength and position^19,23^. Due to climate change, Shaw and Miyawaki predict increased jet stream velocities^24^, while Hallam et al., mentioned consistent latitude increases in the Northern Hemisphere Jet Stream (NHJET)^25^. Woollings et al., linked tropical warming to jet stream changes^26^, while Alizadeh and Babaei analyzed seasonal variability in the North Pacific Ocean Jet Stream (NPOJET). In another study, they recognized that there were not many changes in the height of the NPOJET during the summer between 1979 and 2020^27^.

The position and nature of jet streams can be changed by many other phenomena. Anthropogenic warming will shift both hemispheres’ jet streams poleward^28^. Some other studies have demonstrated that storm track activity influences jet stream movements, consequently altering weather patterns^14,29–31^. Jet streams are crucial for transferring moisture, weather patterns, and atmospheric energy, influencing global climate dynamics^32,33^. In each hemisphere, there are polar and equatorial jet streams. Generally, the jet streams in the Northern Hemisphere have been considered one of the most significant meteorological phenomena^19,34^.

Jet streams also display strong year-to-year variability, affected by complicated processes and wide-ranging connections that shape their seasonal and geographical features. Recent studies have found that the summertime East Asian jet changes have increased by an impressive 140%, which is connected to changes in the temperature of the Atlantic sea surface and mid-Pacific circulation patterns^35^. Meanwhile, the North Atlantic jet also shows yearly changes influenced by thermal fronts similar to the Gulf Stream and the balance of thermal winds^36^. These fluctuations are strongly modulated by large-scale climate oscillations such as El Niño, the North Atlantic Oscillation, and the Pacific-North American index, which amplify or dampen jet stream variability across seasons^37–39^. The evolving patterns of winter teleconnections, influenced by greenhouse gas emissions, and the intensified effects along the polar front jet underscore the intricate dynamics at play^38,40^. These indices signify the effects of prolonged alterations in atmospheric circulation, which are recognized to indicate climate shifts^41,42^.

Beyond climate impacts, jet streams have practical implications that benefit aviation by reducing route times and costs. In 1952, a Pan American flight utilized the jet stream to cut travel time^24^. Since they cause clear-air turbulence^5^, the jet stream potential for energy harnessing using wind turbines is also being explored^43^. Jet streams are also essential in forming and moving atmospheric rivers (ARs), which are narrow and concentrated bands of moisture in the Earth’s atmosphere^44^. As the guiding currents of ARs, jet streams determine their strength, duration, and pathways, directly affecting precipitation distribution and extreme weather events^15,45–48^. Therefore, this research examines the seasonal and monthly temporal and spatial variations and the changes in altitude levels of jet stream occurrences in NA and the NPO from 1984 to 2023 and their interaction with atmospheric temperature changes, considering their significance in various fields.

## Results

**Fig. 1:**
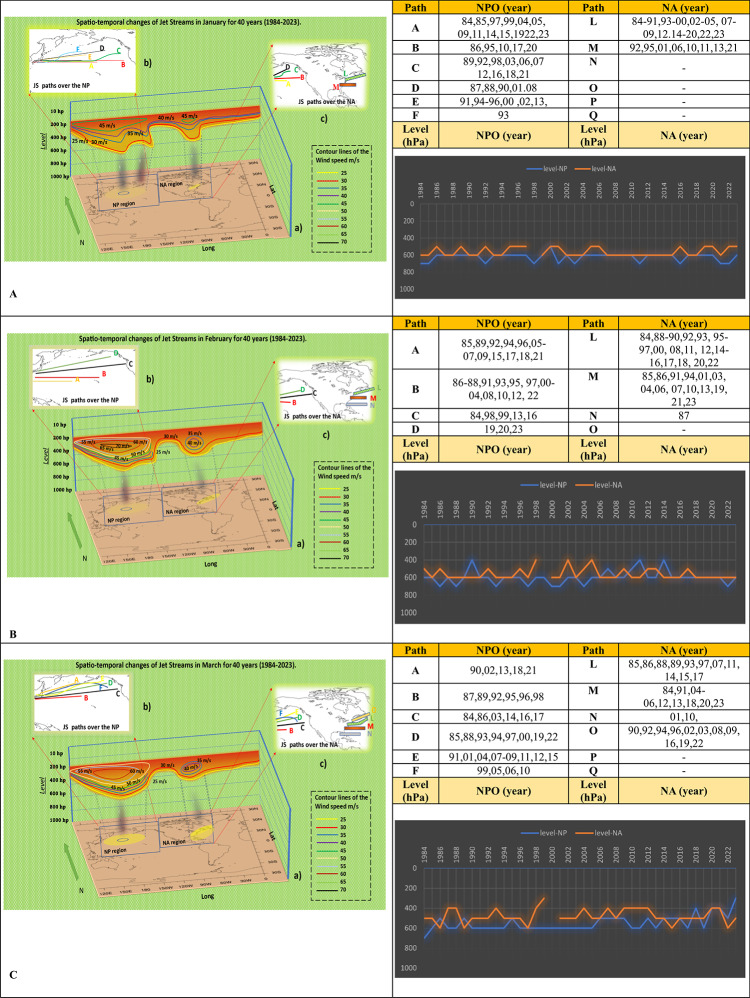
Spatio-temporal Changes of NAJET and NPOJET in January (**A**), February (**B**), and March (**C**) over four decades. [The lines in Figure B show the jet stream trajectories in the NPO.] [The lines in Figure C indicate the direction of jet streams in the western and eastern parts of NA.] [In Figure A, the colored lines as isotach indicate the connecting points of equal wind speed, determining the atmospheric-level activities of jet stream and the regions affected by jet streams] [The vertical black shading indicates regions of maximum jet stream suction, where its mechanism is most intense and extends its influence to lower atmospheric levels] [The tables reveal the annual activities of jet streams on each path in January, February, and March.] [The histograms display the trend of changes in the atmospheric levels of jet streams from 1984 to 2023 in both the NA and NPO regions, with the blue line representing the NPO and the orange line for NA]. The absence of a year in the table and the broken line in the diagram indicate that no jet stream activity was detected during that specific month/year based on the synoptic analysis maps.

The study reveals that NAJET in January are active along two distinct pathways, L and M, at different atmospheric elevations of 500 and 600 hPa. These streams have a maximum speed of 45 m/s. The most significant frequency is associated with route L at the 600 hPa level. NPOJET originate on six paths and develop at altitudes ranging from 700 to 500 hPa. Some researchers, however, suggest that they can appear between 400 and 100 hPa^13^. The peak frequency is noted for route A at the 600 hPa level. Notably, the discontinuities visible in most diagrams, such as the one in January of 1998, correspond to months in which no jet stream cores were detected. To validate this finding, the full atmospheric column from 700 to 200 hPa was carefully examined for these months. Despite this assessment, no jet stream activity was observed, confirming that the absence in the diagrams reflects the lack of jet stream occurrences. (Fig. 1A).

The analysis for February reveals that NAJET are generated in three orientations at altitudes ranging from 600 to 400 hPa. Compared to January, they follow a southward trajectory, with the most notable occurrences associated with route L at the 600 hPa level. In contrast, NPOJET maintain consistent strength, flowing in four distinct directions at altitudes ranging from 700 to 400 hPa, with the most significant prevalence again associated with route A and the 600 hPa level. During February, the rate of change in the direction and speed of jet streams accelerated, reaching velocities of 70 m/s over the NPO and 40 m/s in EPNA. The time chart analysis shows that the jet stream level in NA saw multiple increases and even vanished in 1999, in the middle of the analyzed period. However, in the NPO, the frequency of jet stream occurrences rose by an average of 200 hPa (Fig. 1B).

In March, NAJET manifest in four distinct directions L, M, N, and O at altitudes from 300 to 600 hPa. Their origin has shifted farther south to the Gulf of Mexico, with pathways L and O at the 500 hPa level showing the highest frequency. Meanwhile, the NPO sees jet streams forming along six routes, from A to F, spanning altitudes from 300 to 700 hPa, with the most significant activity on route D at 600 hPa. Analysis over decades reveals that while the atmospheric pressure was consistently around 600 hPa in the earlier two decades, the latter two showed variations up to 100 hPa. The slope of isotach lines suggests significant speed changes, peaking at 60 m/s in the NPO and 40 m/s in NA during March. Notably, no jet stream formation occurred in NA in March 2020 (Fig. 1C).

**Fig. 2:**
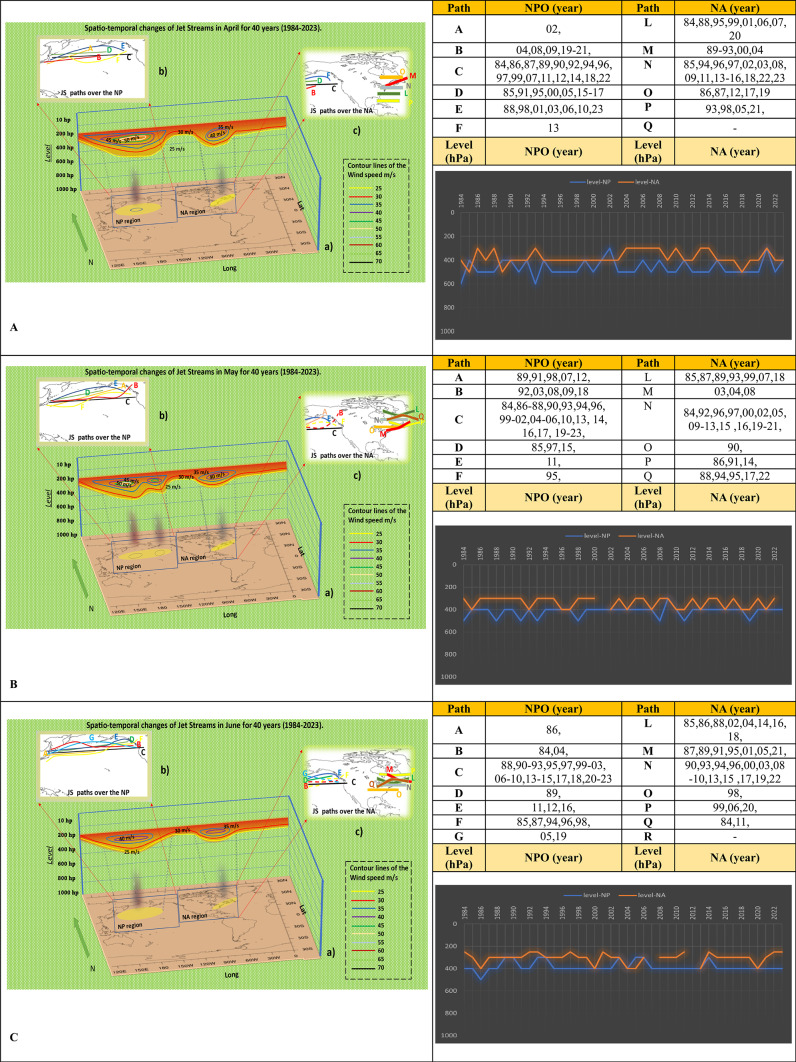
Spatio-temporal Changes of NAJET and NPOJET in April (**A**), May (**B**), and June (**C**) in four decades. [The lines in figures “b” show the Jetstream trajectories in the NPO] [The lines in figures “c” indicate the direction of jet streams in the west and east of NA] [In the shapes “a” we can see the color lines as Isotach which indicate the connecting points of equal wind speed. It also determines the atmospheric-level activities of jet streams, and the region affected by jet streams] [The vertical black shading indicates regions of maximum jet stream suction, where its mechanism is most intense and extends its influence to lower atmospheric levels] [The tables reveal the annual activities of jet stream in each path in April, May, and June] [The histograms show the trend of changes in atmospheric-level of jet streams from 1984 to 2023 in NA and NPO regions. The blue line is for NPO, and the orange line is for NA]. The absence of a year in the table and the broken line in the diagram indicate that no jet stream activity was detected during that specific month/year based on the synoptic analysis maps.

In the spring months, noticeable changes in the characteristics of the jet stream may be seen after the conclusion of the winter season. In April, there is an evident shift in the trajectory of the jet stream in the eastern region of NA, spanning from lower to higher latitudes. An analogous scenario about the number of jet stream trajectories compared to March has been seen in the NPO. In this month, three linear pathways and three curvilinear paths were seen. Evidently, the highest velocity of jet streams in NPO has reached 50 m/s and has fallen by ten hPa compared to March. However, in NA, its value has stayed constant at 40 m/s. Jet streams often occur at 500 hPa for NPO and 400 hPa for NA. In the NPO, the atmospheric level activities of the jet stream have seen a variation of 300 hPa, and this variation has shown an increase compared to March. The variation in NA has been 200 hPa for 13 years, from 1990 to 2003.Jet streams most often travel paths C and N in a straight direction in both areas (Fig. 2A).

In May, both areas had an equal number of jet stream occurrence routes, with each region having six paths that were either straight or wavy. They have moved back towards higher latitudes in the eastern part of NA and paths C and N are known as the main paths. Two cores at a pressure of 50 hPa were detected at 300 hPa above the NPO. The altitude variations in May compared to April are noteworthy due to a noticeable increase in altitude during that month. The variability of the jet stream intensity in the NPO is comparatively lower than in previous months and typically occurs at an altitude of 400 hPa. The atmospheric level of variability for the NA area was initially minimal throughout the first half of the analyzed period, mostly around 300. Since 2002, it has shown a consistent pattern of variation; however, jet stream occurrences were absent in the NA area in 2001 and 2023 (Fig. 2B).

June is one of the months in which the jet stream’s path varies significantly. NPO has seven routes, and NA has six routes; nevertheless, paths C and N are still the main routes. The maximum core velocity of the jet streams has weakened in both regions and has been active at high levels in the atmosphere. The fluctuation of elevation levels during these 40 years has been low in both areas. In the NPO, jet streams typically move at 400 hPa, whereas in the eastern parts of NA, the typical altitude for jet streams is 300 hPa. During the years 2007 and 2012, there were no notable incidents involving jet streams. Generally, the jet streams in June are more orderly than in previous months, displaying fewer geographical and altitude level changes. Jet streams undergo fewer changes and fluctuations as they ascend to higher altitudes, resulting in more stable and predictable patterns (Fig. 2C).

**Fig. 3:**
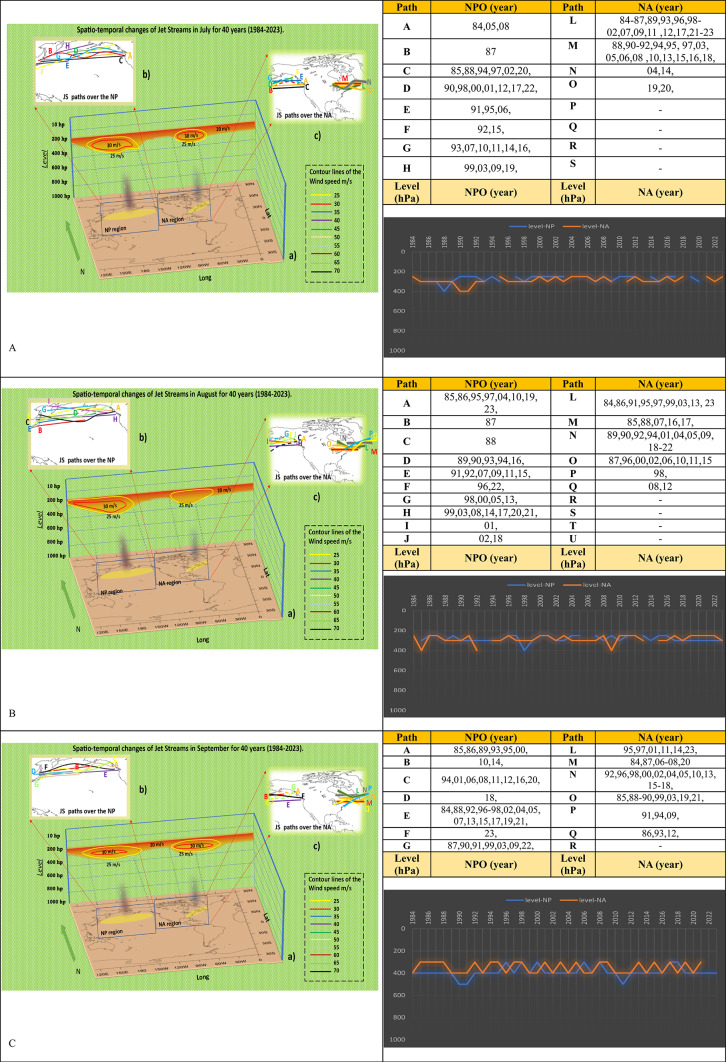
Spatio-temporal Changes of NAJET and NPOJET in July (**A**), August (**B**), and September (**C**) in four decades. [The lines in figures “b” show the Jetstream trajectories in the NPO] [The lines in figures “c” indicate the direction of jet streams in the west and east of NA] [In the shapes “a” we can see the color lines as Isotach which indicate the connecting points of equal wind speed. It also determines the atmospheric-level activities of jet streams, and the region affected by jet streams] [The vertical black shading indicates regions of maximum jet stream suction, where its mechanism is most intense and extends its influence to lower atmospheric levels] [The tables reveal the annual activities of jet stream in each path in July, August, and September] [The histograms show the trend of changes in atmospheric-level of jet streams from 1984 to 2023 in NA and NPO regions. The blue line is for NPO, and the orange line is for NA]. The absence of a year in the table and the broken line in the diagram indicate that no jet stream activity was detected during that specific month/year based on the synoptic analysis maps.

Investigating the fluctuations of jet streams over each month can provide insights into their transitions between cold and warm seasons. Further examination of the analysis reveals that the alterations that occur during the warmer season are substantial. Long-term investigations revealed in July that the jet streams’ primary velocity had decreased significantly compared to the preceding months. The utmost velocity of the jet stream has reduced to 30 m/s, and its strength has also diminished. The number of jet stream paths in the NPO has surpassed eight, compared to four in the eastern region of NA. The paths were generally lengthy and irregular; identifying the primary route would become more difficult as the number of routes expanded. L and M are the principal jet stream trajectories in eastern NA. The main atmospheric level of the jet streams in both regions is 250 and 300 hPa. The data reveals that the absence of a jet stream in July has occurred in seven instances in the NPO and four cases in the East of NA throughout the study period (Fig. 3A).

The deceleration of the jet stream in the eastern region of NA persisted throughout August. The maximum velocity recorded is 25 m/s, while in the NPO, it has maintained a consistent pace of 30 m/s, the same as in July.: The quantity of pathways is continuing to grow. This month, the NPO had ten distinct patterns of jet stream passage in NPO and six tracks extending towards eastern NA. Both study regions exhibit a significant amount of route fluctuation. The length of jet streams in the NPO has reached its maximum and extends from East Asia to West NA. Jet streams typically occur at altitudes between 250 and 300 hPa in the atmosphere, although, in some instances, they have been seen to shift to lower altitudes. In August, referring to the long-term study, the NPO experienced three years, and the NA experienced two years in which the jet stream was absent (Fig. 3B).

Since September, jet stream activity has appeared at lower levels of the atmosphere. The jet streams of this month have tended to operate at the levels of 300 and 400 hPa with a maximum speed of 30 m/s in both studied areas and path N can be considered the main path of jet streams in eastern NA. The movement of the paths has assumed a straight direction, resulting in a reduction in their undulation. Since there have been two cases of height drops in the NPO, the jet streams above this region tend to descend to lower levels after passing the cold months, which is also true for most of the previous months. The graph of the changes in the altitude levels of the jet stream event shows that the NPOJETs are more orderly than in the eastern part of NA, and the jet streams tend to fluctuate a lot in most years for the eastern part of NA. Except for 2022 in the east of NA, there has been no month without jet stream events during the September study period in both regions (Fig. 3C).

**Fig. 4:**
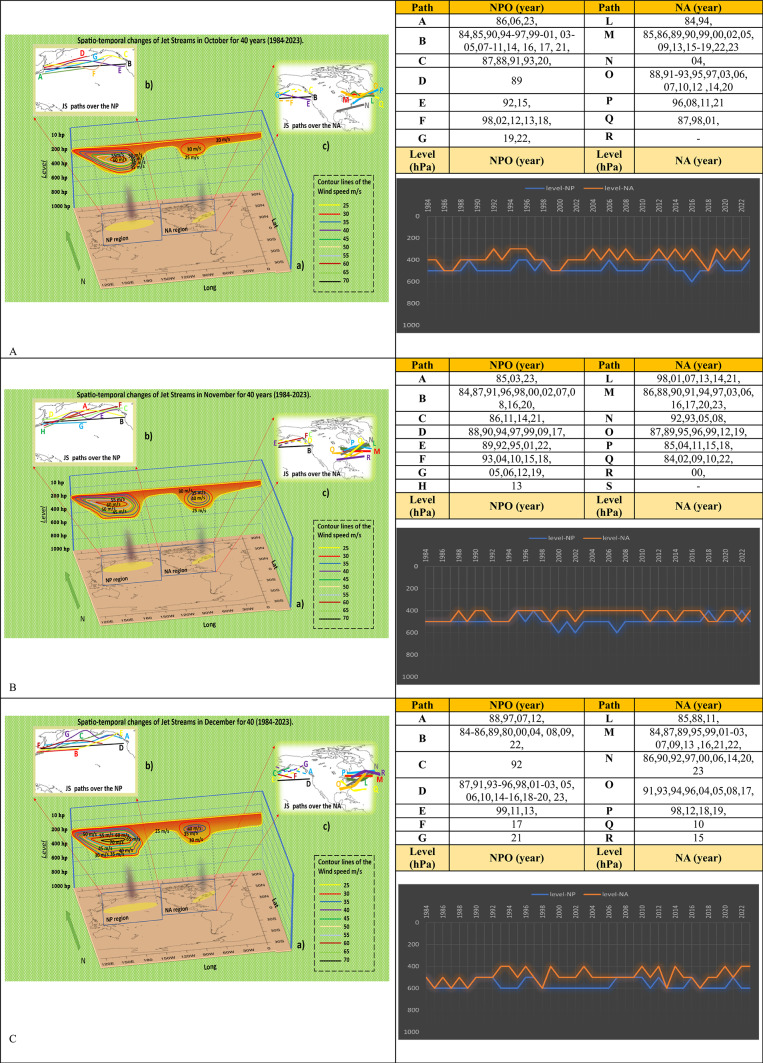
Spatio-temporal Changes of NAJET and NPOJET in October (**A**), November (**B**), and December (**C**) in four decades. [The lines in figures “b” show the Jetstream trajectories in the NPO] [The lines in figures “c” indicate the direction of jet streams in the west and east of NA] [In the shapes “a” we can see the color lines as Isotach which indicate the connecting points of equal wind speed. It also determines the atmospheric-level activities of jet streams, and the region affected by jet streams] [The vertical black shading indicates regions of maximum jet stream suction, where its mechanism is most intense and extends its influence to lower atmospheric levels] [The tables reveal the annual activities of jet stream in each path in October, November, and December] [The histograms show the trend of changes in atmospheric-level of jet streams from 1984 to 2023 in NA and NPO regions. The blue line is for NPO, and the orange line is for NA]. The absence of a year in the table and the broken line in the diagram indicate that no jet stream activity was detected during that specific month/year based on the synoptic analysis maps.

The shift from the warm to the cold season would significantly alter the dynamics and atmospheric currents. This transmission is vividly demonstrated in the maps presented in Fig. 4. The primary and easily observable characteristic of jet streams is their velocity. In October, there was a considerable rise in the compression of isotach lines, which indicate wind speed, compared to September. These adjustments suggest a doubling in pace compared to the prior month. A velocity of 60 m/s demonstrates the formidable strength of the NPOJET. In the eastern region of NA, the changes are not substantial, and the highest feasible velocity of active jet streams in this area is 30 m per second, which is half the speed of the NPOJET. Paths B, M, and O serve as the primary route for jet streams in both regions. The atmospheric height stability of the NPOJET is more significant than that in the eastern part of NA. The histogram reveals that the variability in the vertical fluctuations of the NPO in October is much lower than that of the jet streams in the eastern region of NA. However, it is only sometimes continuous, and during the last decade, this regularity has slightly diminished (Fig. 4A).

November brings about a unique set of characteristics for the jet streams. They maintain a consistently high velocity in the NPO and intensify in the eastern part of NA. The velocity has increased by up to 10 m per second. In November, the NAJET exhibited an identifiable characteristic of several jet stream pathways that span a considerably large extent and length. In October, routes B and M were identified as the most actively frequented jet stream routes in both investigation areas. However, the number of jet stream pathways has since expanded in both regions. The altitude at which the jet stream occurs this month is relatively high. During the analyzed period, there is a noticeable variation in the NPOJET’s level, particularly around 600 hPa. Conversely, the NAJET remains consistently stable during this timeframe. Typically, throughout November, the NPOJET and ENAJET exhibit movement at 500 and 400 hPa (Fig. 4B).

The observable changes toward the end of the fall season mark a significant progression in our understanding of jet stream behavior. These changes can be examined in the context of December. During this month, jet streams shifted toward higher latitudes, and their velocities increased to as much as 70 m/s in the atmosphere over the NPO. The isotach lines showed significant compression, indicating substantial fluctuations in the jet streams over the NPO. The number of jet stream trajectories in both regions was considerable, reaching seven. The flow direction of EPNAJET exhibited significant variations within a small geographical region. NPOJET fluctuated between 500 and 600 hPa, while jet streams over NA varied between 400 and 600 hPa. Graph analysis indicates that the altitude of jet streams has increased over time, with jet streams operating at higher atmospheric levels (Fig. 4C).

Table 1 presents a detailed summary of jet stream behaviour over various months across four decades in two regions: the NPO and NA, indicated by blue and orange asterisks. In the NPO, February experienced the least jet stream pathways, while August recorded the highest number. Lower-altitude jet streams are active in winter, whereas higher-altitude jet streams dominate in summer. Peak speeds are recorded in December and February, and jet stream absence is more common during summer. Wavy jet stream paths appear in all months except February, while direct paths are present year-round. Fluctuations in activity amplitude occur at different atmospheric levels: 100 hPa in the warm season, 200 hPa starting in late summer, and 300 hPa in February and April.

**Table 1:**
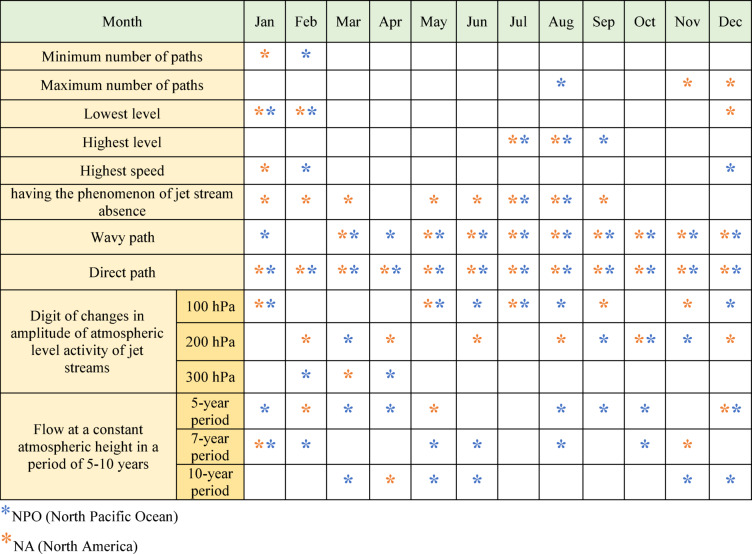
Information and attributes of the jet stream during four decades in the NA and NPO.

The maximum number of jet stream paths in NA is observed in November and December, while the minimum occurs in January. The frequency and types of path movements (wavy or direct) resemble those in the NPO, and peak speeds are recorded in January. Jet stream’s absence has been recorded in nearly all months, excluding fall and April. Variations in activity amplitude are observed at 100 hPa and 200 hPa year-round, although, at 300 hPa, fluctuations are most pronounced in March.

**Fig. 5:**
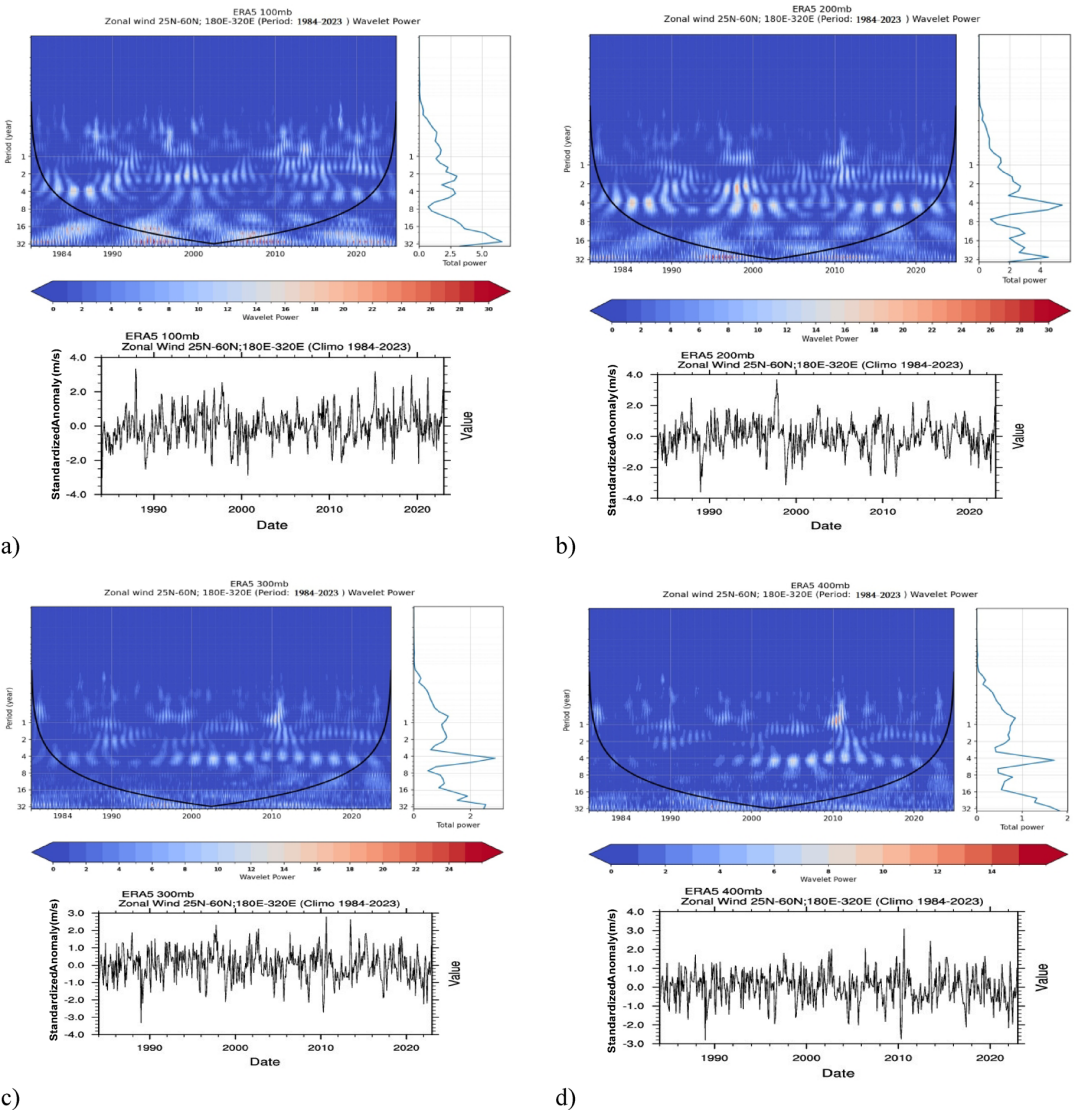
Wavelet Power Spectrum Analysis of Zonal Wind Anomalies (1984–2023). Panels (**a**)–(**d**) show the wavelet power spectrum and anomaly time series for zonal wind at (**a**) 100 mb, (**b**) 200 mb, (**c**) 300 mb, and (**d**) 400 mb, using ERA5 data from 25°N to 60°N, 180°E to 320°E. This figure highlights periodicities (1–32 years), with significant regions (95% confidence) marked by black contours as the cone of influence, revealing dominant cycles. Standardized anomalies in the time series range from -4 to 4, indicating interannual variability across each level.

To investigate the performance of the cyclical behavior of jet streams across four pressure levels (100 to 400 millibars), a wavelet power spectrum analysis of regional wind anomalies was conducted using the ERA5 dataset over the study period, with a focus on the study region (Fig. 5). The analysis reveals a diverse range of cycles across these atmospheric levels. At the 100-hPa level, short-term cycles are observed throughout the study period, exhibiting a lack of distinct regularity and generally indicating weaker jet stream activity in this upper atmospheric layer. As we descend to the 200-hPa level, a noticeable strengthening of jet stream cycles emerges, particularly those with periods of 4 years and extending beyond 5 years, suggesting a gradual intensification over time. Further down into the lower troposphere at the 300- and 400-hPa levels, cycles of approximately 5 years and less intense 7- and 10-year cycles become prominent, especially from 2000 onward. The right-hand panel of the maps provides a more precise visualization of these temporal shifts, emphasizing the evolving patterns. The jet stream’s variability over time is reflected in standardized anomaly time series, which fluctuate between − 4 and 4 m per second. This range underscores significant interannual variability across each pressure level, highlighting the dynamic nature of jet stream behavior. These findings suggest that the jet stream’s cyclical patterns may be influenced by short-term atmospheric processes at higher altitudes and longer-term climatic oscillations at lower tropospheric levels, offering valuable insights into the atmospheric dynamics within the studied region.

**Fig. 6:**
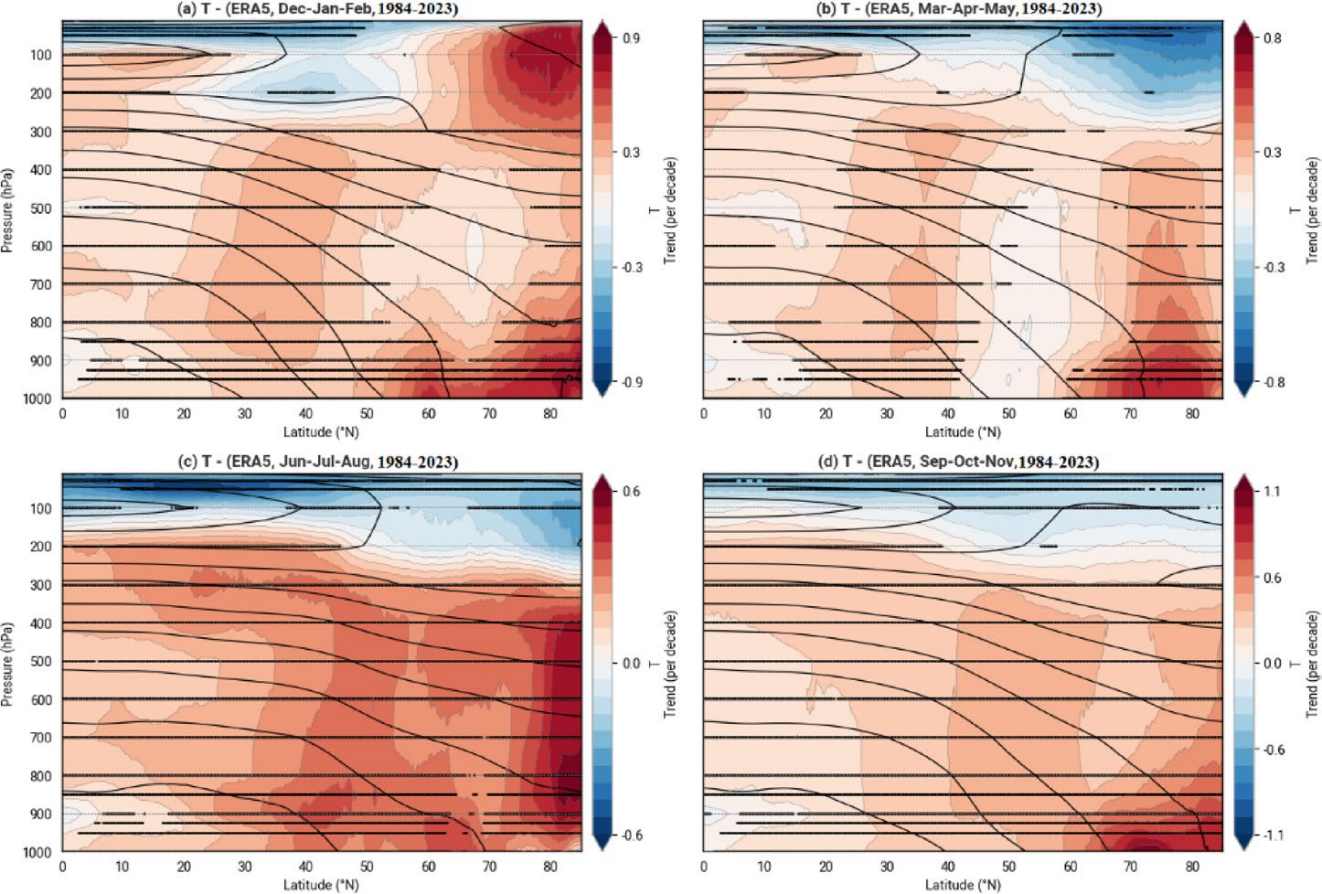
Relative Trends of Temperature (T) as a function of latitude and pressure from ERA5 in the study area (1984–2023) determined using the Modified Mann–Kendall Test to account for autocorrelation. (**a**) Dec-Jan-Feb, (**b**) Mar-Apr-May, (**c**) Jun-Jul-Aug, (**d**) Sep-Oct-Nov. The color scale represents the percentage change in temperature per decade. Contour lines depict the long-term mean temperature (K).

Figure 6 illustrates the linear trends of temperature as percentage changes per decade across four seasons—(a) Dec-Jan-Feb, (b) Mar-Apr-May, (c) Jun-Jul-Aug, and (d) Sep-Oct-Nov—based on ERA5 reanalysis data from 1984 to 2023. The color scale, ranging from − 1.1% to + 1.1%, highlights consistent warming, peaking at + 1.1% in SON, followed by + 0.9% in DJF, + 0.8% in MAM, and + 0.6% in JJA. Notably, JJA experienced the most significant temperature increase among all seasons. Across all seasons over the four decades, a marked temperature rise near the Earth’s surface in the polar region (60–80°N) is observed, averaging 1 K per decade (4 K over 40 years), likely due to ice melt in the Arctic, reducing solar reflectivity and increasing absorption. In DJF, a peak decrease in temperature at the tropopause near the northern pole is evident, accompanied by a downward intrusion of cold weather into mid-latitudes (30–50°N). In contrast, during MAM, JJA, and SON, temperatures decrease from 200 hPa upward, reflecting a cooling trend in the tropopause. These trends were assessed using the Mann–Kendall test, adapted through the Hamed-Rao method to ensure robustness at a 95% confidence level.

**Fig. 7:**
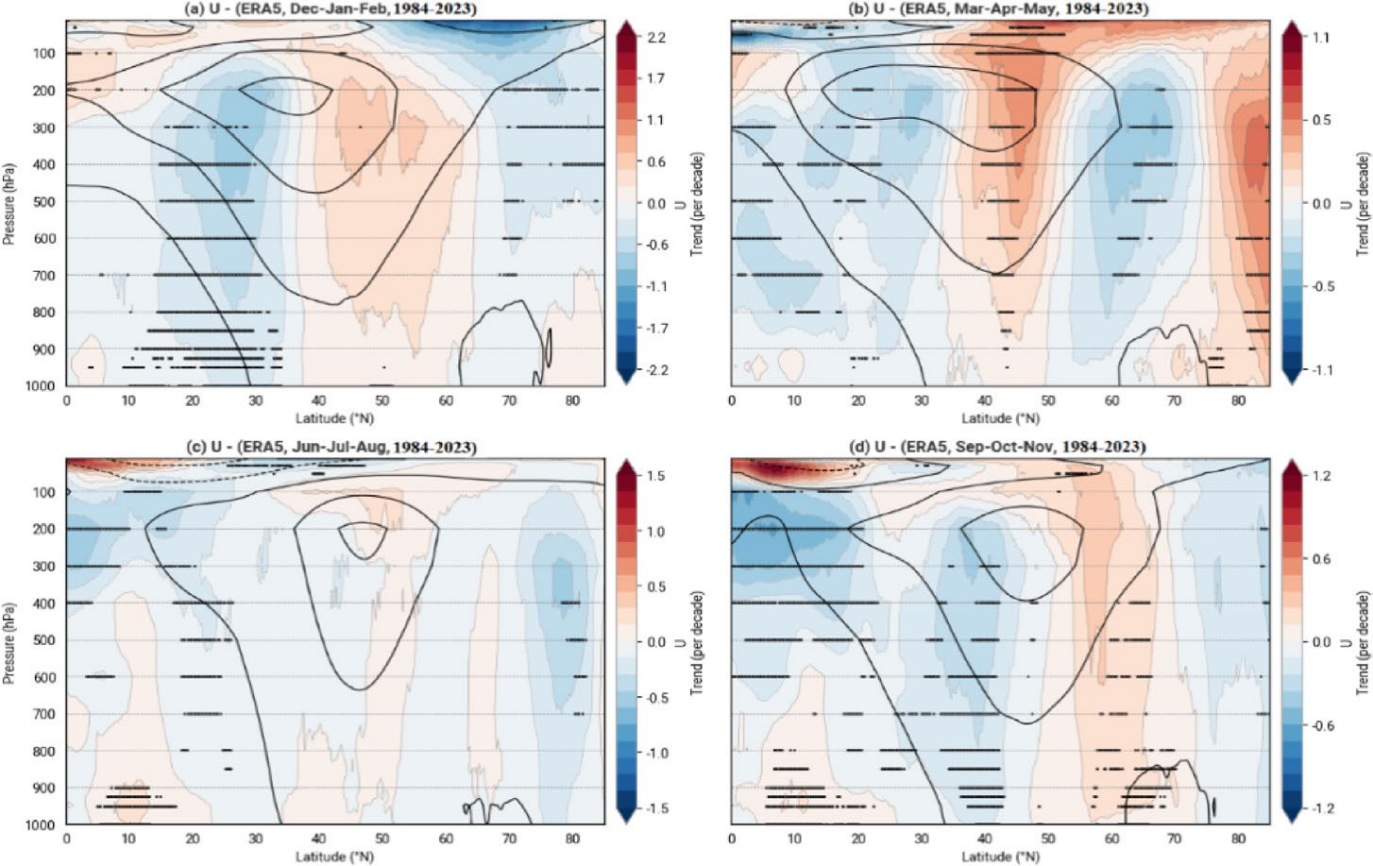
Relative Trends of Zonal Wind (U) as a function of latitude and pressure from ERA5 in the study area (1984–2023) determined using the Modified Mann–Kendall Test to account for autocorrelation. (**a**) Dec-Jan-Feb, (**b**) Mar-Apr-May, (**c**) Jun-Jul-Aug, (**d**) Sep-Oct-Nov. The color scale represents the percentage change in zonal wind per decade. Contour lines indicate the long-term mean zonal wind (m/s), highlighting the jet stream structure. Positive trends (red) suggest strengthening, while negative trends (blue) indicate weakening.

Continuing the analysis of the pathways and altitudinal changes in jet stream movements, Fig. 7 clarifies the seasonal variations observed over the past four decades. Blue-colored regions indicate an increase in wind speed, while red areas signify a decrease. The mean altitude of the jet stream core activity is approximately 200 hPa, though its influence extends down to 700 hPa. Long-term examinations reveal that the jet streams have shifted approximately 10 degrees toward northern latitudes, moving poleward, with this displacement being evident across all seasons except JJA. Referring to previous maps, which identified the 40–50°N latitude band as the primary pathway for jet stream activity, the core of jet stream activity in SON and MAM has shifted to higher altitudes. It appears that should atmospheric rivers align with jet stream patterns, Canada may experience an increased frequency of atmospheric river events. However, these changes are less pronounced for MAM in northern Canada, with a noted reduction in jet stream occurrences above 60°N.

## Discussion

Global warming is anticipated to impact the dispersion of mass (as well as the pressure) inside the atmosphere, influencing the intensity and location of the jet streams. Identifying the characteristics of and investigating the monthly and seasonal changes of jet streams over an extended period is crucial. Jet streams have a complex structure and temperament, exhibiting discontinuity in time and location. They are also associated with substantial variations in wind speed, height, and direction of movement. Multiple studies have examined jet streams; however, in NA, there has not been a thorough examination that addresses the frequency, routes, features of high-speed cores, path characteristics, and fluctuations in altitude. This work obtained monthly average data for Vwnd and Uwnd and T from ERA5, ERA-Interim, and NCEP/NNCAR. The data have a spatial resolution of 0.25 × 0.25, 0.75 × 0.75 and 2.5 × 2.5 degrees, respectively, covering 1984–2023. Then, maps were generated and used in the investigations. To further explore these dynamics, the relative seasonal trends of T and Uwnd were analyzed from 1000 to 10 hPa across the atmosphere, employing the Mann–Kendall test, modified through the Hamed-Rao method to account for autocorrelation, at a 95% confidence level, providing clear insights into the decadal changes in jet stream behavior and associated temperature patterns. Complementing this analysis, wavelet power spectrum maps were generated to examine the cyclic behavior of zonal wind anomalies at four pressure levels (100 mb, 200 mb, 300 mb, and 400 mb) using ERA5 data from 1984 to 2023, focusing on the study region. These maps, with power intensity displayed on a color scale and significance assessed at a 95% confidence level, revealed dominant periodicities, such as 4–10-year cycles, potentially linked to large-scale atmospheric phenomena. The inclusion of the cone of influence delivered the reliability of the results by highlighting areas unaffected by edge effects, while accompanying time series of monthly anomalies provided additional context for interpreting the observed cycles.

The results showed two regions where jet streams significantly impact the climate of NA: one above the NPO and the other above EPNA. Seasonally, in the NPO, the highest number of jet stream paths occurs in the summer, while in EPNA, it occurs in the fall. The lowest number of jet stream paths for both regions is in the winter.

Jet streams are active at lower atmospheric levels in winter, while in summer, they are active at higher atmospheric levels. Additionally, the NOPJET move at a lower altitude than those over EPNA. However, as the jet streams rise in altitude in summer, this difference almost diminishes. In terms of movement patterns, throughout the year, except for February and January, when the jet streams move relatively straight, both straight and wavy movements can be observed. The altitude fluctuation range of jet stream activity is between 100 and 300 hPa, with significant fluctuations in February and April and the least in December, January, and May to August. Over the 40-year study period, there have been cyclical periods without altitude fluctuations in the jet streams. There are three fixed cycles of 5, 7, and 10 years during which the jet streams move at a relatively constant altitude. This feature has been observed in all months except July. Generally, the NPOJET tend to move straight and without waves. Regarding speed, analyses showed that jet streams reach their lowest speed and strength in summer. With the onset of the cold season, their speed increases significantly, reaching up to 70 m per second, which indicates a direct relationship between their altitude and speed.

The results also showed that winter and spring jet streams over the NPO tend to move at higher atmospheric altitudes over time, partially indicating a reduction in their fluctuation. On the other hand, winter and fall jet streams over EPNA tend to be active at lower altitudes, indicating increased waviness and significant volatility in their paths. Geographically, jet stream flows can affect the region from the Gulf of Mexico to Northern Hudson Bay, especially in March and April. Due to the dynamic mechanism of the jet stream, this impact can absorb a significant amount of moisture from the Gulf of Mexico and transport it towards the provinces of Quebec and Newfoundland in Canada. As explained above, this decrease in altitude can lead to increased occurrences of phenomena such as atmospheric rivers originating from the south, impacting NA’s eastern and northeastern regions because there is an inverse relationship between the decrease in altitude and the amount of moisture in the atmosphere^49–51^.

The wavelet power spectral analysis revealed diverse jet stream cycles across four pressure levels (100 to 400 mb) in the study region. At 100 hPa, short-term cycles lack regularity, indicating weak jet stream activity. At 200 hPa, 4- to 5-year cycles show a clear strengthening, while at 300 and 400 hPa, 5-year cycles dominate, with weaker 7- and 10-year cycles emerging after 2000. Standardized anomalies fluctuate from -4 to 4 m/s, highlighting significant interannual variability and suggesting influences from short-term upper-level processes and longer-term lower tropospheric oscillations.

Seasonal analyses of temperature and wind patterns over the past four decades reveal significant changes. Jet streams have migrated toward higher latitudes, and during spring and autumn, their core activity has shifted to higher atmospheric altitudes. Temperature maps indicate that regions experiencing warming have also seen zonal wind speed increases of 0.5 to 1.5 m per second per decade. These stronger winds are likely to intensify individual storms, potentially leading to more severe weather events, such as atmospheric rivers. Temperature increases have been most pronounced in polar regions, likely due to changes in the ozone layer due to the manipulation of greenhouse gases, snowmelt altering surface color, consequently reducing albedo, and increasing the absorption of solar radiation.

According to Stendel et al., a region of intense warming is expected near the tropopause in response to increased latent heat in a warming atmosphere. As a result, the polar temperature gradient in the upper atmosphere increases, strengthening westerly winds at higher levels and altering vertical atmospheric stability, particularly in tropical regions. They noted that it remains unclear which of the two enhanced warming regions—surface warming in the Arctic or upper-level warming near the tropopause—has a greater influence on jet stream dynamics under climate change conditions and how these effects vary by season, region, and prevailing climatic context^34^. In this study, we investigated these dynamics on a seasonal basis. Williams et al., demonstrated that increased vertical wind shear, driven by the vertical temperature gradient at the tropopause with the lower atmosphere, enhances jet stream shear^52^. Our study found that cooling in the lower stratosphere or tropopause contributes to increased jet stream speeds. These vertical shear patterns underscore the need for regional analyses of jet streams. In this paper, we identified two distinct regions and examined the characteristics of their jet streams.

## Methods

A scale including a longitude range of 0–120 degrees west and a latitude range of 0–90 degrees north was chosen to delineate the jet streams that impact the atmosphere over NA and the NPO. Subsequently, data on Uwnd and Vwnd and T parameters for the statistical period spanning from 1984 to 2023 were acquired from the NCAR/NCEP monthly reanalysis data available on the website of the United States National Oceanic and Atmospheric Administration (NOAA), ERA-Interim data and finally ERA5 as the best sets of reanalyzed weather data available^53–55^, and the required maps were produced. These maps covered several atmospheric altitudes and were generated monthly for 40 years from the 200–700 hPa level. While most previous studies on jet streams have focused on the 300–200 hPa levels, our analysis of the maps revealed that in many cases the jet stream cores extend downward to around 550 hPa. Based on this observation, we analyzed a broader vertical range from 700 to 200 hPa to better capture their full structure. In general, 3360 maps were generated and analyzed to study the variations of jet stream at the seven atmospheric levels (700, 600, 500, 400, 300, 250, and 200) with 2.5*2.5, 0.75*0.75 and 0.5*0.5 degrees of horizontal resolution for 40 years.

The pathways of the jet streams were determined by first applying a wind speed threshold of 25 m/s, which is commonly used to define the presence of jet stream cores. Based on this criterion, synoptic maps were generated for each month and season, and the central axis of the jet stream core was then traced to identify its trajectory. This approach allowed us to capture both the jet streams’ spatial pathway and vertical structure across different temporal scales. Frequencies were calculated to determine the occurrence of the jet stream in various months of the year. The dominant patterns of the NAJET and NPOJET were identified in terms of their spatial and temporal occurrences each month and at different atmospheric altitude levels. A 3D Hoff-Müller diagram graphically represented the jet stream’s speed in the study area. The design employed longitude as a constant variable, while the latitude and speed of the jet stream were represented as variable data. To investigate the changes in the jet streams at different atmospheric levels better, the longitude where the jet stream center is located and its latitude and altitude as variables have been considered. Afterward, the jet stream profiles were prepared. At the end of the temporal and spatial changes, the jet stream 's altitude levels, the maximum activity cores, and the main routes during the statistical period (1984–2023) were investigated.

To investigate seasonal changes in air temperature and zonal wind, this study extracted monthly temperature and zonal wind component data from the ERA5 reanalysis dataset from 1980–2023. The spatial domain encompassed latitudes from 0° (equator) to 90°N and longitudes from 180°W to 20°W. Longitudinal averages were computed for pressure levels ranging from 1000 to 10 hPa, disaggregated by latitude.

For seasonal analysis, four time periods were defined: winter (December–February), spring (March–May), summer (June–August), and autumn (September–November). In each season, monthly data corresponding to the respective months were selected and aggregated into annual seasonal averages. Long-term trends for each parameter were evaluated using the modified Mann–Kendall test, adjusted for autocorrelation, at a significance level of α = 0.05. Trend slopes were calculated using Sen’s slope estimator and expressed in units of change per decade.

To visualize the results, four seasonal maps were generated, with pressure levels on the vertical axis and latitude on the horizontal axis. Seasonal averages were depicted using contour lines, while trend slopes (per decade) were illustrated with color-coded maps on the same coordinate system. Statistically significant trends were highlighted with dotted lines. This set of maps provides a clear, quantitative depiction of long-term changes in temperature and zonal wind, enabling season-to-season comparisons.

To further investigate the cyclic behavior and temporal periodicity of zonal wind associated with jet stream dynamics, a wavelet power spectrum analysis was conducted. Monthly anomaly data for zonal wind were extracted from the ERA5 reanalysis dataset for the period 1984–2023, focusing on the spatial domain of 25°N to 60°N latitude and 180°E to 320°E longitude. This analysis was performed at four pressure levels 100 mb, 200 mb, 300 mb, and 400 mb to capture variations in jet stream behavior across different altitudes. The wavelet power spectrum was generated for each pressure level, where the power intensity was represented on a color scale, with time and period. The cone of influence was included to highlight regions potentially affected by edge effects, supplying the reliability of the analysis. Statistical significance was assessed at a 95% confidence level, providing robust identification of dominant cycles. Additionally, the corresponding time series of monthly zonal wind anomalies were plotted below each wavelet power spectrum to provide context for the periodicity analysis. This methodology enabled the identification of significant periodicities, such as 4–10-year cycles, which may be linked to large-scale atmospheric phenomena and facilitated the comparison of cyclic behavior across different atmospheric levels.

## Data Availability

The data used in this paper have been prepared from databases extracted from the National Oceanic and Atmospheric Administration (NOAA) and the European Centre for Medium-Range Weather Forecasts (ECMWF) sites: 1- https://psl.noaa.gov/data/gridded/data.ncep.reanalysis.html. 2- https://cds.climate.copernicus.eu/cdsapp#!/dataset/reanalysis-era5-pressure-levels-monthly-means?tab=form.

## Code availability

Standard plotting tools were used and no custom code or algorithms were central to this study.
